# Comparison of O-polysaccharide and hemolysin co-regulated protein as target antigens for serodiagnosis of melioidosis

**DOI:** 10.1371/journal.pntd.0005499

**Published:** 2017-03-30

**Authors:** Apinya Pumpuang, Susanna J. Dunachie, Phornpun Phokrai, Kemajittra Jenjaroen, Kitisak Sintiprungrat, Siriphan Boonsilp, Paul J. Brett, Mary N. Burtnick, Narisara Chantratita

**Affiliations:** 1 Department of Microbiology and Immunology, Faculty of Tropical Medicine, Mahidol University, Bangkok, Thailand; 2 Department of Clinical Pathology, Faculty of Medicine, Vajira Hospital, Navamindradhiraj University, Bangkok, Thailand; 3 Mahidol-Oxford Tropical Medicine Research Unit, Faculty of Tropical Medicine, Mahidol University, Bangkok, Thailand; 4 Centre for Tropical Medicine & Global Health, Nuffield Department of Medicine, University of Oxford, Oxford, United Kingdom; 5 Department of Microbiology and Immunology, University of South Alabama, Mobile, Alabama, United States of America; University of California San Diego School of Medicine, UNITED STATES

## Abstract

**Background:**

Melioidosis is a severe disease caused by *Burkholderia pseudomallei*. Clinical manifestations are diverse and acute infections require immediate treatment with effective antibiotics. While culture is the current diagnostic standard, it is time-consuming and has low sensitivity. In endemic areas, inaccessibility to biosafety level 3 facilities and a lack of good serodiagnostic tools can impede diagnosis and disease surveillance. Recent studies have suggested that O-polysaccharide (OPS) and hemolysin co-regulated protein 1 (Hcp1) are promising target antigens for serodiagnosis of melioidosis.

**Methodology/Principle findings:**

We evaluated rapid ELISAs using crude antigens, purified OPS and Hcp1 to measure antibody levels in three sets of sera: (i) 419 serum samples from melioidosis patients, Thai and U.S. healthy donors, (ii) 120 serum samples from patients with other bacterial infections, and (iii) 423 serum samples from 200 melioidosis patients obtained upon admission and at 12 and 52 weeks post-recovery. We observed significantly higher antibody levels using the crude antigen prepared from wild type *B*. *pseudomallei* K96243 compared to that of an OPS-mutant. The areas under receiver operator characteristics (AUROCCs) for diagnosis were compared for individual Hcp1-ELISA or OPS-ELISA or combined Hcp1/OPS-ELISA. For Thai donors, AUROCCs were highest and comparable between the Hcp1-ELISA and the combined Hcp1/OPS-ELISA (0.95 versus 0.94). For U.S. donors, the AUROCC was highest for the combined Hcp1/OPS-ELISA (0.96). Significantly higher seropositivity was observed in diabetic patients compared to those without diabetes for both the Hcp1-ELISA (87.3% versus 69.7%) and OPS-ELISA (88.1% versus 60.6%). Although antibody levels for Hcp1 were highest upon admission, the titers declined by week 52 post-recovery.

**Conclusions/Significance:**

Hcp1 and OPS are promising candidates for serodiagnosis of melioidosis in different groups of patients. The Hcp1-ELISA performed better than the OPS-ELISA in endemic areas, thus, Hcp1 represents a promising target antigen for the development of POC tests for acute melioidosis.

## Author summary

Melioidosis, caused by *Burkholderia pseudomallei*, is a life-threatening infection endemic in tropical countries. Definitive diagnosis of melioidosis relies upon bacterial culture which requires suitable laboratory facilities and reliable antibody testing. To obtain an effective target antigen for use in a simple point-of-care (POC) test, rapid ELISAs using crude *B*. *pseudomallei* antigen preparations or purified O-polysaccharide (OPS) and hemolysin co-regulated protein (Hcp1) were compared using serum samples from three large collections obtained from melioidosis patients and patients with other bacterial infections. We detected high levels of antibodies to Hcp1 and OPS in serum from melioidosis patients upon admission and showed that anti-Hcp1 levels declined post-recovery. When serum samples from endemic areas were tested, the performance of the Hcp1-ELISA and combined Hcp1/OPS-ELISA were higher than the OPS-ELISA. When serum from non-endemic areas was tested, the combined Hcp1/OPS-ELISA gave the highest performance. Both the OPS- and Hcp1-based ELISAs were useful for detection of antibodies in various groups of patients including diabetics. Since anti-Hcp1 titers in melioidosis patient serum were higher than anti-OPS titers, Hcp1 is an attractive candidate for further development of a rapid POC test for use in endemic areas.

## Introduction

Melioidosis is a severe infectious disease caused by the Gram-negative environmental bacterium, *Burkholderia pseudomallei*. It is an under-recognized tropical disease that is a common cause of community-acquired infections in Southeast Asia and northern Australia. It is recognized that melioidosis is a more significant global public health concern than previously thought, with increasing numbers of cases reported in many countries [1]. A recent report estimated the incidence of melioidosis to be 165,000 cases per year worldwide, with a predicted annual mortality of 89,000 [2]. In Thailand, the estimated incidence rate is 12.7 cases of melioidosis per 100,000 people per year and the mortality rate is 43% [3]. Melioidosis is the third most common cause of death from infectious diseases in northeast region after HIV infection and tuberculosis [3]. Up to 80% of patients with melioidosis have one or more risk factors which include diabetes, alcohol use, renal disease, thalassemia, cancer and glucocorticoid therapy [1, 4]. Among these, diabetes is the most common underlying disease with 60% of melioidosis patients being diabetic [1].

*B*. *pseudomallei* is a facultative intracellular pathogen [5] that can invade host cells, escape from phagosomes, survive within the cytosol and spread from cell-to-cell in many organs [6, 7]. These processes are dependent upon virulence-associated type III and type VI secretion systems (T3SS and T6SS) expressed by this pathogen [8, 9]. Lipopolysaccharide (LPS) and capsular polysaccharide (CPS) are additional virulence factors that contribute to the pathogenesis of *B*. *pseudomallei* [10]. The clinical manifestations of melioidosis are diverse and can mimic other infections, ranging from skin and soft tissue infections to acute pneumonia and septicemia frequently resulting in misdiagnosis. Treatment of melioidosis requires immediate administration of ceftazidime or carbapenems, which are generally not used as empirical treatment for other bacterial sepsis [1].

Making an early and accurate diagnosis of melioidosis to guide treatment is critical for reducing patient mortality. The diagnosis of melioidosis and subsequent appropriate treatment depends on culture of *B*. *pseudomallei* from clinical specimens, or evidence of sepsis in people with a high risk of exposure and predisposing factors (e.g. diabetes) for melioidosis. However, identification of *B*. *pseudomallei* by culture is time-consuming (typically 72 hours), has low sensitivity (60%) [11, 12] and requires both experience and stringent laboratory health and safety for this Hazard Group 3 pathogen. Using culture methods, laboratories unfamiliar with *B*. *pseudomallei* frequently misidentify the bacterium as an inconsequential environmental *Pseudomonas* species [13].

An alternative approach to the gold standard of bacterial culture for diagnosis of melioidosis is antigen detection using a monoclonal antibody to *B*. *pseudomallei* capsule as a point-of-care (POC) diagnostic lateral flow assay (LFA). Although rapid and low cost, the LFA only achieves 40% sensitivity in blood of culture-positive patients, limiting its diagnostic utility in acute melioidosis [14]. Quantitative real-time polymerase-chain reaction (qPCR) assay of clinical samples may provide a more rapid result than culture, but has a disappointing sensitivity at 61% in northeast Thailand, especially when performed on blood (sensitivity at 25%) [15]. Additional tests such as latex agglutination assays, immunofluorescence assays or matrix-assisted laser desorption ionization-time of flight mass spectrometry (MALDI-TOF MS) are required to accelerate the identification of positive cultures [16–19].

To improve the time for diagnosis of melioidosis, an indirect hemagglutination assay (IHA) is used to determine antibody titers that are indicative of exposure to *B*. *pseudomallei*. While rapid compared with bacterial culture, the sensitivity and specificity of the IHA in Thailand are low (69.5% and 67.6% respectively) [20]. We recently developed a simpler O-polysaccharide (OPS)-based latex agglutination assay which shows potential for detecting exposure to *B*. *pseudomallei* in individuals from non-endemic areas but lacks specificity in long term residents from endemic regions [20].

A rapid POC serological test with high sensitivity and specificity would be ideal for use in resource-poor areas where melioidosis is endemic. To develop such assays, identification of good serologic markers is critical. It is also important to evaluate whether an assay can differentiate between acute melioidosis and previous infection or exposure. Our recently developed rapid indirect enzyme-linked immunosorbent assay (ELISA) provides a platform for evaluation of different antigen candidates [21]. Among several antigens tested, our studies and others have highlighted the potential of *B*. *pseudomallei* OPS and hemolysin co-regulated protein 1 (Hcp1) as targets for further development of serodiagnostic tests for melioidosis [20–24]. Hcp proteins are both structural components and substrates of T6SSs [24], and in *B*. *pseudomallei* are known to be expressed in vivo [9, 22, 23].

To identify the best candidate for further development of POC, we used rapid ELISAs to measure antibodies to OPS and Hcp1 using our large collections of serum samples from both endemic and non-endemic areas. The aims of this study were 1) to compare the antibody responses measured by ELISA to OPS and non-OPS antigens in sera from melioidosis patients and healthy donors, 2) to develop a rapid ELISA using Hcp1 as the target antigen for antibody detection and then compare the results of Hcp1-ELISA with the OPS-ELISA, 3) to evaluate the diagnostic potential of Hcp1 and OPS for determination of antibody titers in different groups of melioidosis patients, and 4) to evaluate the dynamics of the antibody responses to OPS and Hcp1 over 12 months in individual melioidosis patients by comparing titers during acute infection with the titers observed at 3 and 12 months post-recovery.

## Materials and methods

### Serum samples

Initially, two sets of anonymous human serum samples were used to evaluate the ELISAs as described previously [21]. The first set included 141 on-admission sera from culture-confirmed *B*. *pseudomallei* infected patients who were admitted to Sappasithiprasong hospital, Ubon Ratchathani, northeast Thailand, 188 serum samples obtained from healthy donors from the same area in northeast Thailand and 90 serum samples obtained from healthy U.S. donors (Innovative Research, Novi, MI, USA). The second set was three groups of on-admission anonymous human sera that were used to further evaluate the specificity of the ELISAs. These included the following groups: 1) 20 acid-fast stain positive tuberculosis patients from Chiangrai, north Thailand, 2) 50 culture-proven scrub typhus patients from Udon Thani, northeast Thailand, and 3) 50 culture-proven leptospirosis patients from Udon Thani, northeast Thailand.

To evaluate the diagnostic potential of Hcp1 and OPS antigens for determination of antibody titers in different groups of melioidosis patients, a third set of independent serum samples was used. This set included serum samples obtained from patients with culture-confirmed melioidosis collected a median of 5 days (Interquartile range, IQR 3–6 days, range 2–13 days after admission (N = 200), and at 12 weeks (N = 113) and 52 weeks (N = 110) post-recovery. The patients were recruited in a longitudinal clinical and immunological study at Sappasithiprasong hospital during September 2012-October 2015 [25]. All participants were ≥ 18 years old. All serum samples were stored at -80°C.

### Ethical approval

The study was approved by Ethics Committee of Faculty of Tropical Medicine, Mahidol University (approval number MUTM 2014–079 and MUTM 2012–018), Sappasitthiprasong hospital (approval number 018/2555), and the Oxford Tropical Research Ethics Committee (reference 64–11). Written informed consent was obtained from the participants enrolled in the study.

### Preparation of antigens

Whole-cell (WC) antigen was extracted from the wild type strain *B*. *pseudomallei* K96243 (from a Thai patient in northeast Thailand; expresses type A OPS) and an OPS mutant (*ΔwbiD* K96243) by heating at 80°C for 1 h. The supernatant was used as the antigen described previously [21, 26]. The OPS mutant defective in *wbiD* (BPSL2677) was constructed as described in our previous study [27].

*B*. *pseudomallei* LPS type A was extracted from the select agent excluded strain RR2808 (capsule mutant) using a modified hot phenol method [28, 29]. Purified OPS antigen was then obtained using acid hydrolysis and gel permeation chromatography as previously described [30]. For expression of recombinant Hcp1 (rHcp1) with a N-terminal 6xHis-Tag, the *hcp1* ORF (BMAA0742) was PCR amplified from *B*. *mallei* ATCC 23344 genomic DNA using the Bmhcp1-6HisF (5’-CCCAACGGTCTCACATGGCGGCGCATCATCATCATCATCATCTGGCCGGAATATATCTCAAGG-3’) and Bmhcp1-R1 (5’-CCCAACGGTCTCAAGCTTCAGCCATTCGTCCAGTTTGCGGC-3’) primer pair; BsaI linkers are underlined. The resulting DNA fragment was digested with BsaI and cloned into pBAD/HisA digested with NcoI/HindIII producing plasmid pBADBmhcp1-6HisF. Notably, *B*. *pseudomallei* and *B*. *mallei* Hcp1 proteins are 99.4% identical. Recombinant DNA techniques were conducted as previously described [31]. Oligonucleotide primers were obtained from Integrated DNA Technologies. DNA sequencing was performed by ACGT Inc. For purification of rHcp1, *E*. *coli* TOP10 (pBADBmhcp1-6HisF) was grown to mid log phase in LB broth and protein expression was induced using 0.02% L-arabinose (Sigma). Bacterial pellets were resuspended in B-PER (Pierce) plus Benzonase (Novagen) and Lysozyme (100 μg/ml) and incubated for 20 min at room temperature with gentle agitation. Insoluble material was removed by centrifugation and the resulting supernatant was loaded onto a gravity-fed Ni-NTA agarose (Invitrogen) column. The column was washed with Wash Buffer (50 mM Tris pH 8.0, 300 mM NaCl and 40 mM Imidazole), protein was eluted with Elution Buffer (50 mM Tris pH 8.0, 50 mM NaCl and 300 mM Imidazole) then dialyzed against PBS and loaded onto a gravity-fed His-Pur Cobalt Resin (Thermo Scientific) column. The column was washed with PBS and rHcp1 was eluted with Wash Buffer, dialyzed against PBS, concentrated and stored at 4°C. Protein concentrations were determined using a BCA protein assay kit (Pierce). Endotoxin removal was performed using High Capacity Endotoxin Removal Resin (Pierce). The amount of endotoxin in the rHcp1 preparations was quantitated using a LAL Chromogenic Endotoxin Quantitation Kit (Pierce).

### ELISA

The ELISAs were performed using these following antigens: 1) WC antigen prepared from wild type *B*. *pseudomallei* K96243, 2) WC antigen prepared from an OPS mutant defective in OPS (*ΔwbiD* K96243) antigen, 3) rHcp1 protein, 4) the purified OPS antigen, and 5) OPS antigen in combination with rHcp1 (Hcp1/OPS). The optimal concentration of coating antigen was determined using pooled melioidosis and pooled healthy sera as previously described [21]. Following evaluation for antigen concentration and serum dilution, the plates were prepared for ELISA using the optimized antigen concentration as follows: WC 0.25 μg/ml, OPS 1 μg/ml, Hcp 2.5 μg/ml and OPS/Hcp1 (0.5 μg/ml OPS/1.25 μg/ml Hcp1). The serum samples used for evaluation of the various ELISAs included culture-confirmed melioidosis patients (N = 141), U.S. healthy donors (N = 90), Thai healthy donors (N = 188), tuberculosis patients (N = 20), scrub typhus patients (N = 50) and leptospirosis patients (N = 50) at a dilution of 1:2,000. All ELISAs were performed using a 1:2,000 dilution of horseradish peroxidase-conjugated rabbit antihuman IgG as previously described [21].

### Determination of antibody titers to Hcp1 and OPS

To examine the antibody titers specific to Hcp1 and OPS, ELISAs were performed with a third set of serum samples obtained from acute phase and recovery phase melioidosis patients using undiluted sera and two-fold serial dilution sera at range of 1:125 to 1:2,048,000. The endpoint antibody titer was read at the serum dilution which showed positive OD values of each ELISA. Positive results for individual serum samples were determined using OD cut-off values at specificity of 95%. The antibody titers of individual patients were compared between week 0, week 12 and week 52. Only samples from different time points with two-fold changes in titer were considered as increased or decreased antibody titers.

### Statistical analysis

Statistical analyses were performed using Stata version 12 (StataCorp LP, College Station, TX) and Prism 5 Statistics (GraphPad Software Inc, La Jolla, CA). All data in box plots are presented as 25^th^ and 75^th^ percentile boundaries in the box with the median line within the box; the whiskers indicate the 10^th^ and 90^th^ percentiles. The Mann-Whitney test was used to test the difference of median between different serum groups. Spearman’s rank correlation was used to determine the pairwise correlation coefficient for the pairs of tests [32]. The McNemar test was used to compare the sensitivity between tests. Fisher’s exact test was performed to compare the ELISA results and clinical presentation and outcomes. Differences were considered statistically significant at a p-value < 0.05.

A receiver operator characteristic (ROC) curve was created to monitor the shifting the positive cut off value on true-positive (sensitivity) and false positive (1-specificity) rates. Areas under the ROC curves (AUROCC) were compared using a nonparametric method as previously described by DeLong et al. [33]. The ELISA data of the melioidosis group and Thai donors were evaluated separately from the data of the melioidosis group and U.S. donors using OD cut-off values at specificities of 95%.

## Results

### Comparison of antibodies to *B*. *pseudomallei* wild type and OPS mutant

**Fig 1:**
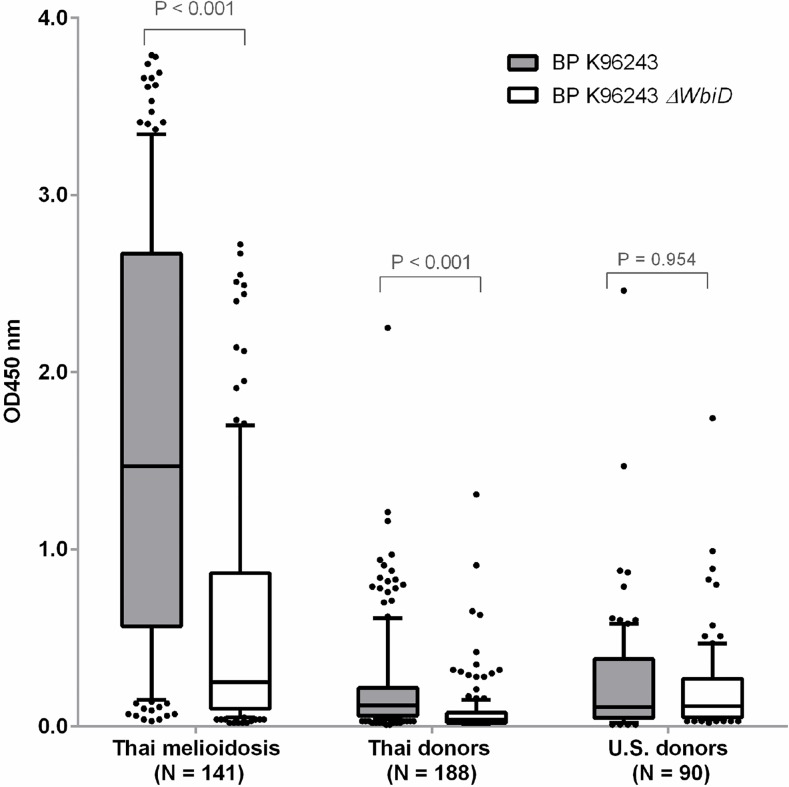
Antibodies to whole cell antigens of *B*. *pseudomallei* K96243 wild type and OPS mutant (*ΔwbiD* K96243). ELISAs were performed on the plates coated with antigens using serum from melioidosis patients, Thai healthy donors and U.S. healthy donors at dilution of 1:2000. Box plots represent 25^th^ and 75^th^ percentile boundaries in the box with the median line within the box; the whiskers indicate the 10^th^ and 90^th^ percentiles. The plots show OD 450 nm for each antigen.

Results from our previous study using a crude antigen ELISA (WC-ELISA) to determine the levels of *B*. *pseudomallei*-specific antibodies in five individual melioidosis patients, to either wild-type (K96243) or an OPS mutant *(*K96243*ΔwbiD*), indicate that OPS appears to be the predominant antigen recognized by human antibodies [21]. In the present study, we expand these experiments to determine the antibody levels in 419 individual sera obtained from melioidosis patients (N = 141), Thai healthy donors (N = 188), U.S. healthy donors (N = 90) using the same WC-ELISA with coating antigens prepared from either the wild type or the OPS mutant (Fig 1). Our results revealed that the median OD value for the melioidosis group was statistically higher compared to Thai donors (*P* < 0.001 for both ELISAs) and U.S. donors (*P* < 0.001 for both ELISAs). The median OD value for the melioidosis group was 5.9 times lower in the OPS mutant-WC-ELISA compared to the wild type-WC-ELISA. Similarly, the median OD value for Thai healthy donors was 3 times lower in the OPS mutant-WC-ELISA in comparison to the wild type-WC-ELISA [median OD 0.04 (IQR 0.02–0.08) versus 0.12 (IQR 0.06–0.22); *P* < 0.001]. In contrast, the median OD value for U.S. healthy donor serum was not significantly different between the two ELISAs [median OD 0.12 (IQR 0.05–0.27) for OPS mutant versus 0.11 (IQR 0.05–0.38) for wild type; P < 0.954]. These findings suggest that OPS is a predominant antigen recognized by antibodies in Thai melioidosis patients and Thai healthy donors sera. This was not the case, however, for sera from U.S. donors. Interestingly, results from the OPS mutant-WC-ELISA also revealed that several melioidosis patients appeared to have high antibody levels to antigens other than OPS.

### Optimization of rapid ELISA for Hcp1

Recently, our group and others have shown that Hcp1 is a promising candidate serodiagnostic marker for melioidosis [22–24]. *B*. *pseudomallei* Hcp1 is a T6SS component that is expressed *in vivo* or under iron-limiting conditions when the organism is grown *in vitro* [22, 24]. To assess the serodiagnostic potential of Hcp1, we developed a rapid ELISA using rHcp1 as the target antigen and compared it with our established OPS-ELISA [21]. The optimal conditions for our Hcp1-ELISA were initially determined using pooled serum from either melioidosis patients or healthy donors. The optimized concentration of rHcp1 for coating wells was 2.5 μg/ml. For the primary antibody incubation step, we used a serum dilution of 1:2000 at room temperature (25°C) for 30 minutes. The assay was standardized throughout the study using these conditions for all serum samples as previously described [21].

### Comparison of antibodies to Hcp1 and OPS in serum samples

For comparison with our previous study using an OPS-ELISA, a total of 539 serum samples were tested in our Hcp1-ELISA [21]. These included on-admission sera from culture-proven melioidosis patients (N = 141), Thai healthy donors (N = 188), U.S. healthy donors (N = 90), tuberculosis patients (N = 20), scrub typhus patients (N = 50) and leptospirosis patients (N = 50) [21]. Quantitative results of OD values in both ELISAs are summarized in Table 1. The median OD of melioidosis patients for the Hcp1-ELISA was higher than that of OPS-ELISA [median OD 3.16 (IQR 2.22–3.40) versus 1.78 (IQR 0.67–3.11); P < 0.001]. The median OD value of the melioidosis group was statistically different from Thai healthy donors, U.S. healthy donors, tuberculosis patients, scrub typhus patients and leptospirosis patients for both ELISAs (P < 0.001 for both ELISAs for all comparisons between melioidosis patients versus each of other groups).

**Table 1:** Results of ELISA for antibodies to Hcp1 and OPS. The results were obtained from OD450 values of sera from melioidosis patients, Thai healthy donors, U.S. healthy donors, tuberculosis patients, scrub typhus patients and leptospirosis patients at dilution of 1:2000.

Serum samples	Number of samples	Median OD 450 nm (IQR)		P-value
		Hcp1-ELISA	OPS-ELISA	
Melioidosis patients	141	3.16(2.22–3.40)	1.78 (0.67–3.11)	P < 0.001
Thai healthy donors	188	0.06 (0.03–0.21)	0.12 (0.04–0.30)	P < 0.001
U.S. healthy donors	90	0.17 (0.07–0.38)	0.10 (0.04–0.29)	P = 0.008
Tuberculosis patients	20	0.03 (0.02–0.04)	0.03 (0.02–0.06)	P = 0.989
Scrub typhus patients	50	0.10 (0.03–0.22)	0.13 (0.06–0.29)	P = 0.229
Leptospirosis patients	50	0.05 (0.03–0.20)	0.07 (0.03–0.19)	P = 0.565

### Correlation between antibodies to Hcp1 and OPS in individual sera

We determined the correlation between individual results of the Hcp1-ELISA and OPS-ELISA using serum samples from melioidosis patients, Thai healthy donors, U.S. healthy donors, tuberculosis patients, scrub typhus patients and leptospirosis patients. The pairwise correlation coefficient (rho) of all serum samples was 0.80, however, the relatedness between antibody response to the Hcp1 and OPS antigens was different between groups of serum samples. The results indicate a strong relatedness only in U.S. healthy donor group (rho = 0.93) but the results of the Hcp1-ELISA and OPS-ELISA were less correlated with the groups of Thai melioidosis patients (rho = 0.38) and Thai healthy donors (rho = 0.60). The correlation coefficients were 0.72, 0.50 and 0.74 for tuberculosis, scrub typhus and leptospirosis patients, respectively.

### Receiver operating characteristics of Hcp1-ELISA, OPS-ELISA and combined Hcp1/OPS-ELISA

**Fig 2:**
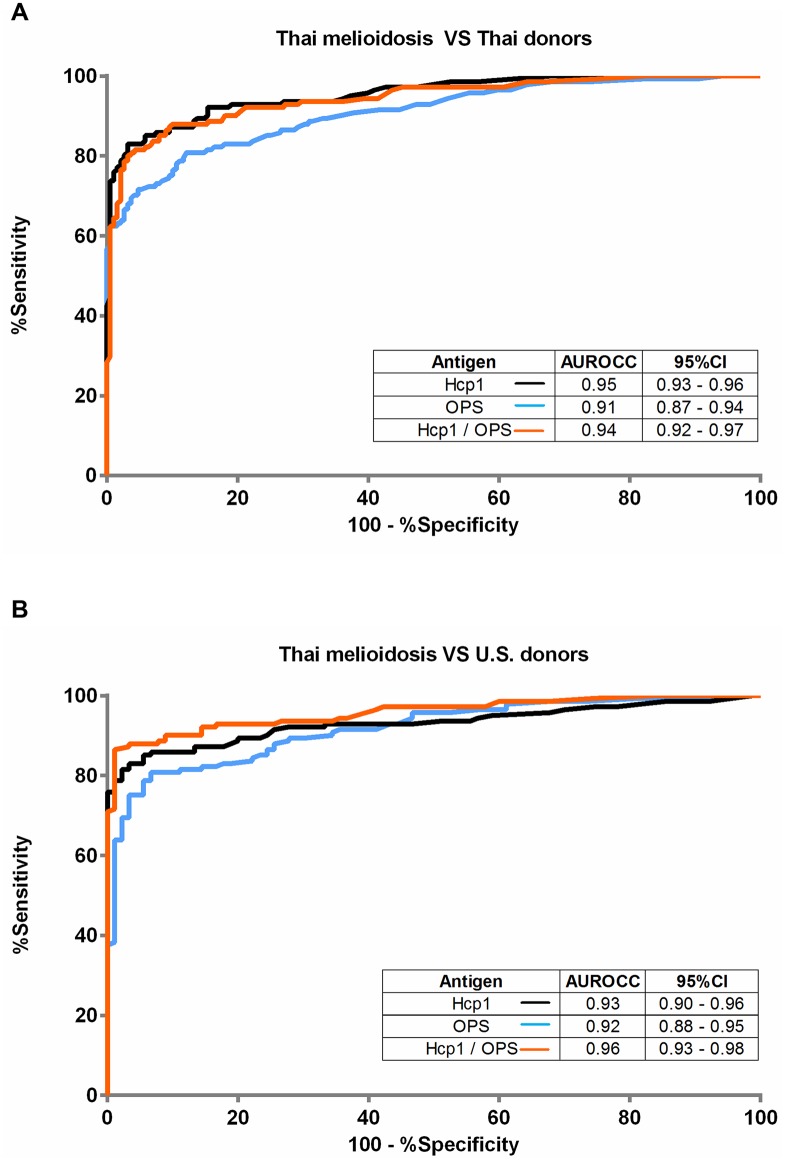
Receiver Operating Characteristics (ROC) plots of the ELISAs of Hcp1, OPS and combined Hcp1/OPS with different groups of serum. (A) Serum form melioidosis patients versus Thai healthy donors, (B) Serum form melioidosis patients versus U.S. healthy donors. The serum samples were diluted 1:2000 for all ELISAs.

We next compared the diagnostic potential of each antigen individually (Hcp1-ELISA or OPS-ELISA) or combined (Hcp1/OPS-ELISA). ROCs were plotted by calculating the sensitivity and specificity of increasing numbers of the true-positive rate and false-positive rate. The results for comparisons of these ELISAs using the melioidosis group and Thai donors are shown in Fig 2A, and those using the melioidosis group and U.S. donors are shown in Fig 2B. When the results of Thai donors were analyzed, the areas under the receiver operator characteristic curves (AUROCCs) for diagnosis of melioidosis were highest and comparable between the Hcp1-ELISA and the combined Hcp1/OPS-ELISA (0.95 versus 0.94, P = 0.153) (Fig 2A). The AUROCC of the OPS-ELISA was significantly lower than that of the Hcp1-ELISA (0.91 versus 0.95, P = 0.001); and lower than that of Hcp1/OPS-ELISA (0.91 versus 0.94, P = 0.003). When the results from the U.S. donors were analyzed (Fig 2B), the AUROCC for diagnosis of melioidosis was highest for the combined Hcp1/OPS ELISA (0.96) and significantly higher when compared to the AUROCC of Hcp1-ELISA (0.93, P = 0.009) and the OPS-ELISA (0.92, P < 0.001). The AUROCC of the Hcp1-ELISA was not significantly different from that of the OPS-ELISA (0.93 versus 0.92, P = 0.353)

### Sensitivity and specificity of Hcp1-ELISA, OPS-ELISA and Hcp1/OPS-ELISA

We further analyzed the sensitivity and specificity of Hcp1-ELISA in comparison to OPS-ELISA and Hcp1/OPS-ELISA using the 539 serum samples described above. To compare the performance of assays, we used an OD cut-off corresponding to a specificity of 95% using Thai healthy donors as controls (OD 1.165) (Table 2). The results demonstrated that the diagnostic sensitivity of the Hcp1-ELISA was significantly higher than that of the OPS-ELISA (83.0% versus 71.6%, P = 0.003). The sensitivity of the Hcp1-ELISA was not significantly different from the sensitivity of combined Hcp1/OPS-ELISA (83.0% versus 81.6%, P = 0.527). The specificity of the Hcp1-ELISA using U.S. healthy donors, tuberculosis patients, scrub typhus patients and leptospirosis patients as non-melioidosis controls were 95.6%, 100%, 98.0% and 100%, respectively. The specificity of the OPS-ELISA using U.S. healthy donors, tuberculosis patients, scrub typhus patients and leptospirosis patients as non-melioidosis controls were 96.7%, 100%, 94.0% and 98.0%, respectively.

**Table 2:** Sensitivity and specificity of Hcp1-ELISA, OPS-ELISA and Hcp1/OPS-ELISA. The assay values were calculated from Thai patients who had melioidosis, tuberculosis, scrub typhus, leptospirosis and Thai healthy donors. The cut-off values with Thai healthy donors as controls were used at specificity of 95% for each assay.

ELISA	Cut-off OD	% Sensitivity (CI)	% Specificity (CI)				
		Thai melioidosis patients (*N* = 141)	Thai healthy donors (N = 188)	U.S. healthy donors (*N* = 90)	Tuberculosis patients (*N* = 20)	Scrub typhus patients (*N* = 50)	Leptospirosis patients (*N* = 50)
Hcp1	1.165	83.0 (75.7–88.8)	96.3 (92.5–98.5)	95.6 (89.0–98.8)	100 (83.2–100)	98.0 (89.4–100)	100 (92.9–100)
OPS	0.875	71.6 (63.4–78.9)	95.7 (91.1–97.8)	96.7 (90.6–99.3)	100 (83.2–100)	94.0 (83.5–98.8)	98.0 (89.4–100)
Hcp1/OPS	1.125	81.6 (74.2–87.6)	95.2 (91.1–97.8)	98.9 (94.0–100)	100 (83.2–100)	88.0 (75.7–95.5)	96.0 (86.3–99.5)

### Antibody titers to Hcp1 and OPS in diabetic and non-diabetic melioidosis patients

**Fig 3:**
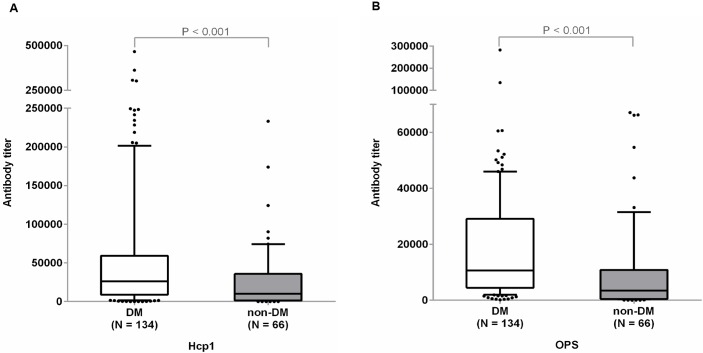
Antibody titers to Hcp1 (A) and OPS (B) in diabetic and non-diabetic melioidosis patients. ELISAs were performed on the plates coated with antigens using undiluted sera and two-fold serially diluted sera from patients. Box plots represent 25^th^ and 75^th^ percentile boundaries in the box with the median line within the box; the whiskers indicate the 10^th^ and 90^th^ percentiles. The plots show antibody titers for each group of patients. The antibody titer was determined by ELISA using cut-off titers at specificity of 95%.

We next investigated whether the use of antibody levels to Hcp1 and OPS as serodiagnostic markers of acute infection in melioidosis patients was influenced by the presence or absence of diabetes (Table 3). The sensitivity and antibody titers to Hcp1 and OPS were determined at week 0 for 200 follow-up patients. At serum dilution 1:2000, we found that the sensitivity of the Hcp1-ELISA for diabetic patients (N = 134) was significantly higher than for non-diabetic patients (N = 66) (87.3% versus 69.7%, P = 0.004). Similarly, the sensitivity of the OPS-ELISA for diabetic patients was significantly higher than for non-diabetic patients (88.1% versus 60.6%, P < 0.001). The median antibody titers for Hcp1 and OPS were significantly higher in diabetic patients compared to non-diabetic patients (P < 0.001 for both Hcp1 and OPS) (Fig 3). The median antibody titers for Hcp1 in diabetic patients was 26,322, (IQR 8,898–59,420) and non-diabetic patients was 10,327 (IQR 1,250–35,842). The median antibody titers to OPS in diabetic patients was 10,657 (IQR 4,487–29,157) and in non-diabetic patients was 3,499 (483.8–10,863).

**Table 3:** Sensitivity of ELISA for antibodies to Hcp1 and OPS in different groups of melioidosis patients. The results were obtained from OD450 values of sera from melioidosis patients at serum dilution of 1:2000. The cut-off values with Thai healthy donors as controls were used at specificity of 95% for each assay.

Group of patients	No. of patients (%)	Hcp1-ELISA			OPS-ELISA		
		No. of positive	Sensitivity (%)	P-value	No. of positive	Sensitivity (%)	P-value
DM	134 (67.0)	117	87.3	**0.004**	118	88.1	**<0.001**
Non-DM	66 (33.0)	46	69.7		40	60.6	
Bacteremia	105 (52.5)	87	82.9	0.654	88	83.8	0.085
Non-bacteremia	95 (47.5)	76	80.0		70	73.7	
Died	64 (32.0)	50	78.1	0.629	50	78.1	0.91
Survived	134 (67.0)	111	82.8		106	79.1	
Unknown	2 (1.0)	2	100		2	100	

Bold represents statistical significance

### Antibody titers to Hcp1 and OPS in melioidosis patients with bacteremia

**Fig 4:**
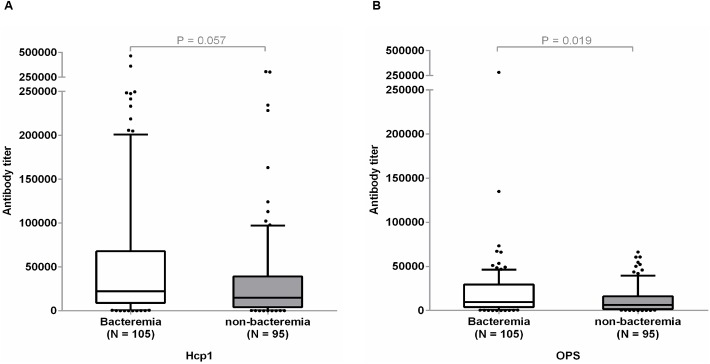
Antibody titers to Hcp1 antigen (A) and OPS antigen (B) in melioidosis patients with bacteremia and non-bacteremia patients. ELISAs were performed on the plates coated with antigens using undiluted sera and two-fold serially diluted sera from patients. Box plots represent 25^th^ and 75^th^ percentile boundaries in the box with the median line within the box; the whiskers indicate the 10^th^ and 90^th^ percentiles. The plots show antibody titers for each group of patients. The antibody titer was determined by ELISA using cut-off titers at specificity of 95%.

We next compared the antibody titers for Hcp1 and OPS in melioidosis patients with or without bacteremia. The levels of Hcp1- or OPS-specific antibodies were determined at week 0 in 200 follow-up patients (Table 3). Using a serum dilution of 1:2000, the sensitivity of the Hcp1-ELISA for patients with bacteremia (N = 105) was not significantly different from that of the patients without bacteremia (N = 95) (82.9% versus 80.0%, P = 0.654). The sensitivity of the OPS-ELISA for patients with bacteremia was not statistically different from patients without bacteremia (83.8% versus 73.7%, P = 0.085). Although the median antibody titer for Hcp1 was not significantly different between bacteremic and non-bacteremic patients (median 22,108, IQR 9,045–66,612 versus median 14,769, IQR 4,154–39,255, P < 0.057), the median antibody titer for OPS was higher in bacteremia patients compared to non-bacteremia patients (median 9,636, IQR 4,150–28,886 versus median 6389, IQR 1817–16106, P = 0.019) (Fig 4).

### Antibody titers for Hcp1 and OPS in melioidosis survivors and non-survivors

**Fig 5:**
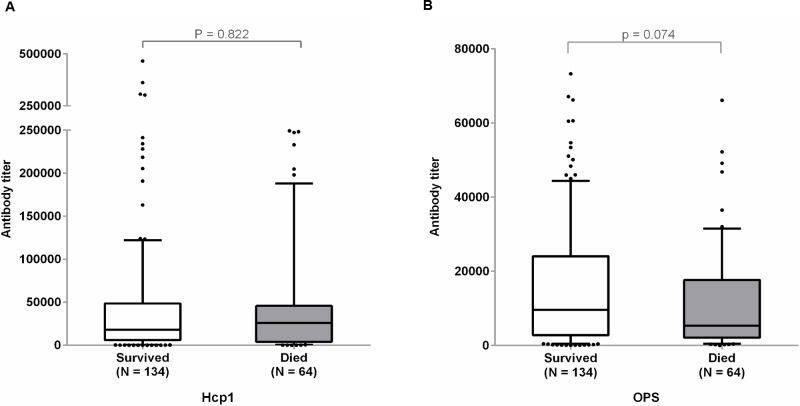
Antibody titers to Hcp1 antigen (A) and OPS antigen (B) in melioidosis patients who survived or died. ELISAs were performed on the plates coated with antigens using undiluted sera and two-fold serially diluted sera from patients. Box plots represent 25^th^ and 75^th^ percentile boundaries in the box with the median line within the box; the whiskers indicate the 10^th^ and 90^th^ percentiles. The plots show antibody titers for each group of patients. The antibody titer was determined by ELISA using cut-off titers at specificity of 95%.

The sensitivities of the Hcp1-ELISA and OPS-ELISA were determined using on-admission (week 0) serum collected from 198 melioidosis patients whose survival status was available (Table 3). The sensitivity of the Hcp1-ELISA for non-survivors (N = 64) was not significantly different from survivors (N = 134) (78.1% versus 82.8%, P = 0.629). The sensitivity of the OPS-ELISA for non-survivors was not different from the patients who survived (78.1% versus 79.1%, P = 0.91). The median antibody titers for Hcp1 in 134 patients who were survived was 18,035 (IQR 6,022–48,678) which was not significantly different from the median antibody titer for 64 non-survivors 25,873 (IQR 4,086–45,678); P = 0.822) (Fig 5). The median antibody titer for OPS in survivors was 9,588 (IQR 2,793–24,096) which was higher than the median of non-survivors 5,330 (IQR 2,114–17,670) but was not significantly different (P = 0.074).

### Detection of antibodies to Hcp1 and OPS in melioidosis patients during acute infection and following recovery

We next investigated whether the antibodies to Hcp1 and OPS might be useful serodiagnostic markers during acute infection and/or following recovery from melioidosis. The specific antibodies were determined in 423 archived serum samples obtained from 200 melioidosis patients recruited in our recent longitudinal study [25]. Using a serum dilution of 1:2000, the percentage of positive serum samples in the Hcp1-ELISA was 81.5% (163/200), 82.3% (93/113) and 81.8% (90/110) at week 0, week 12 and week 52, respectively. The percentage of positive serum samples in the OPS-ELISA was 79.0% (158/200), 85.8% (97/113) and 88.0% (88/110) at week 0, week 12 and week 52, respectively.

**Fig 6:**
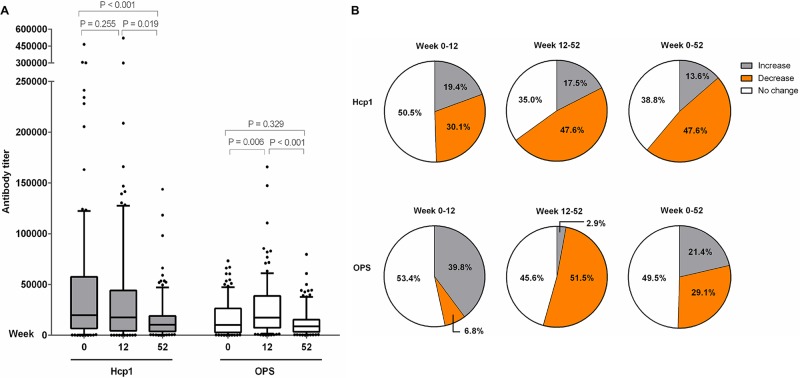
Longitudinal analysis of antibody titers for Hcp1 and OPS in serum samples of 103 melioidosis patients who were survived within one year. The antibody titer was determined at week 0, week 12 and week 52 by ELISAs using cut-off titers at specificity 95%. (A) serum antibody titers, (B) percent of serum samples with increased, decreased and no change in antibody titers between week 0 and 12, between week 12 and week 52, and between week 0 and week 52.

To compare the antibody levels, we determined the endpoint antibody titers to Hcp1 and OPS in 103 individual patients who survived one year after acute infection (Fig 6). The median titer of melioidosis patients for the Hcp1-ELISA at week 0 was not different from that at week 12 [median 19,792 (IQR 6,654–57,596) versus 17,677 (IQR 4,327–44,079); P = 0.255], but the titer was significantly lower at week 52 [median 10,427 (IQR 3,731–19,046); titers of week 12 versus week 52, P < 0.019; week 0 versus week 52, P < 0.001] (Fig 6A). The results of individual patients for Hcp1 are shown in S1 Fig. Of the 103 patient samples tested, 49 (47.6%) showed decreased antibody titers for Hcp1 at week 52 compared to week 0 and 12 while only 31 (30.1%) showed decreased antibody titers for Hcp1 at week 12 compared to week 0 (Fig 6B). However, 14 (13.6%), 20 (19.4%) and 18 (17.5%) of patients had increased titers at week 52 compared to week 0, week 12 compared to week 0, and week 52 compared to week 12, respectively. We found 40 (38.8%), 52 (50.5%) and 36 (35.0%) of patients had no change in antibody titers at week 52 compared to week 0, week 12 compared to week 0, and week 52 compared to week 12, respectively.

In contrast, the median titer of melioidosis patients for OPS-ELISA increased at week 12 compared to week 0 [median 10,192 (IQR 2,783–26,502) versus 17,464 (IQR 7,346–38,834); P = 0.006] but decreased at week 52 [median 8,848 (IQR 3,293–15,359); P <0.001 for comparison between week 12 and week 52] (Fig 6A). The results of individual patients are shown in S2 Fig. The median titer was not different between week 0 and week 52 (P = 0.329). Of a total 103 patients, the number of patients that had decreased antibody titer for OPS at week 52 compared to week 0 was 30 (29.1%), at 12 compared to week 0 was 7 (6.8%), decreased titer at week 52 compared to week 12 was 53 (51.5%) (Fig 6B). However, 22 (21.4%), 41 (39.8%) and 3 (2.9%) of serum samples showed increase titer at week 52 compared to week 0, week 12 compared to week 0 and week 52 compared to week 12 respectively. We found 51 (49.5%), 55 (53.4%) and 47 (45.6%) of the patients had no change in antibody titer at week 52 compared to week 0, week 12 compared to week 0 and week 52 compared to week 12, respectively.

### Correlation of antibodies to Hcp1 and OPS in individual patients after recovery

**Fig 7:**
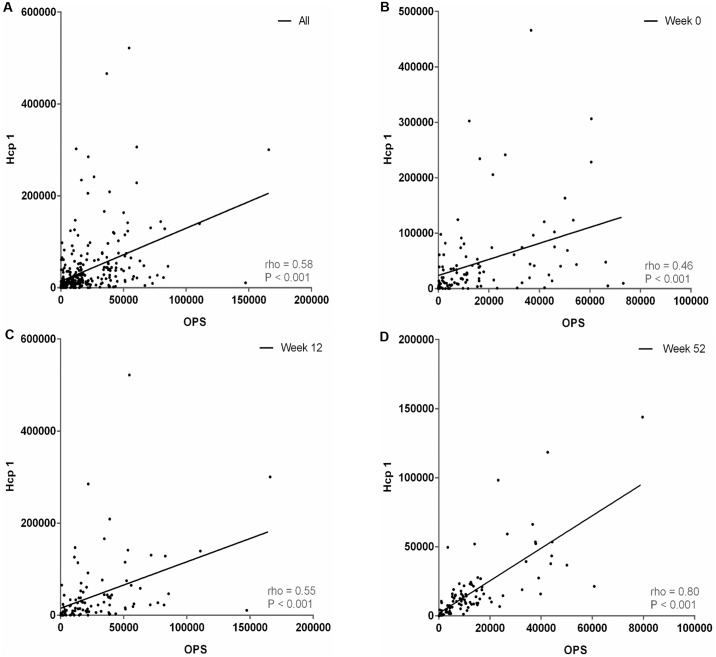
Scatterplots of individual antibody titers to Hcp1 and OPS in serum samples of 103 melioidosis patients who were survived within one year. (A) Sera from all time points, (B) sera of week 0, (C) sera of week 12 and (D) sera of week 52. For each plot, the relationship between variables as determined by linear regression (dotted line), Spearman correlation (correlation coefficient, rho) and P-value are shown.

We next determined the correlation between the individual results from the Hcp1-ELISA and OPS-ELISA conducted using serum samples from 103 follow-up melioidosis patients who had survived at one year after admission (Fig 7). The pairwise correlation coefficient (rho) of all serum samples was 0.58 (P < 0.001). The relatedness between the antibody responses against Hcp1 and OPS was different for the 0, 12 and 52 week sample sets. The rho values at week 0 and week 12 were only 0.46 (P < 0.001) and 0.55 (P < 0.001), respectively. A stronger correlation (rho = 0.80, P < 0.001) was observed with the serum samples collected at week 52.

## Discussion

Melioidosis is a potentially fatal disease that is more widely distributed globally than previously recognized [2, 34]. A rapid and reliable POC serological test would be particularly useful as a tool for serodiagnosis and for seroprevalence studies in highly endemic regions as well as in countries where melioidosis is underreported. In the present study, we used a rapid ELISA platform to assess the serodiagnostic potential of various candidate target antigens. Our results showed that consistent with previous studies, antibodies specific for *B*. *pseudomallei* OPS were predominant in melioidosis patient sera. Interestingly, our WC-ELISA using both a wild type *B*. *pseudomallei* strain and an OPS mutant demonstrated that the median OD value for Thai healthy donors group was significantly lower in the OPS mutant-WC-ELISA compared to the wild type-WC-ELISA. The data suggested that the OPS-specific antibodies might also contribute to the high rate of seropositivity in healthy individuals from endemic areas and may influence the specificity of the assay. In contrast, the median OD value for U.S. healthy donor serum was not significantly different between the two ELISAs. A possible explanation for this finding is that individuals in Thailand and other endemic regions may be previously exposed to environmental *Burkholderia* species that express Type A OPS (e.g. *B*. *thailandensis*) whereas U.S. healthy donors would not. [35, 36].

Hcp1 is a major virulence factor that plays a critical role in the intracellular lifestyle of *B*. *pseudomallei*. Results of this study are consistent with our previous study [37] demonstrating that Hcp1 is immunogenic and is recognized by serum from melioidosis patients. We and others have demonstrated that Hcp1 is expressed at a low level when *B*. *pseudomallei* is cultured *in vitro*, but is produced at a high level *in vivo* within an intracellular environment [24, 37, 38]. Our findings suggest that the detection of antibodies to Hcp1 in a high percentage of melioidosis patients upon admission is likely to reflect infection with *B*. *pseudomallei* rather than non-infective exposure. In addition, since Hcp1 expressed by *B*. *pseudomallei* (and *B*. *mallei*) is structurally different than *B*. *thailandensis* Hcp1, it is likely that seropositivity to this protein antigen will be less prevalent than seropositivity to OPS in healthy donors in endemic areas.

Results of ROC analyses revealed that the Hcp1-ELISA had a significantly higher AUROCC than the OPS-ELISA when serum from Thai healthy donors was used as a control. While the OPS-ELISA alone may not be ideal for detection of acute infections in endemic areas, our data suggest that it may be more useful when combined with Hcp1 for use in non-endemic regions such as the USA. In support of these results, we demonstrated that the median OD value of Hcp1-ELISA in the melioidosis group was significantly higher than the median OD value of the OPS-ELISA. Our results also indicated that antibodies to Hcp1 are significantly elevated in patients with *B*. *pseudomallei* infections. The pairwise correlation coefficient for the results of the two ELISAs for all of the serum samples was high (0.80), however, the relatedness between antibody response to the Hcp1 and OPS antigens was lower in the patient group compared with the two healthy control groups. It is possible that the immune pathways activated by polysaccharide and protein antigens are different among individuals. Hcp1 is a T-cell dependent protein antigen while OPS is a carbohydrate that can induce humoral immune responses via a T-cell independent pathway. A recent report demonstrated that Hcp1 can bind to the surface of host antigen-presenting cells, which may contribute to its immunogenicity by inducing high antibody titers in melioidosis patients [24].

The diagnostic performance of Hcp1-ELISA for antibody detection using the first set of serum samples including 141 Thai melioidosis, 188 Thai healthy donors and 90 U.S. healthy controls showed a significant improvement over the conventional IHA. Using the same serum set, the Hcp1-ELISA had 83% sensitivity and 96% specificity compared with the IHA (sensitivity 69.5% and specificity 67.6%) in our previous study [20]. The results obtained from a second set of 200 melioidosis patient serum samples confirmed the high sensitivity (82%) at the time of admission. Our findings are consistent with a previous report by Lim and colleges [24] which demonstrated that anti-Hcp1 IgG titers in 20 melioidosis patient serum samples were significantly higher compared to serum from 20 healthy controls. In addition, an Hcp1-ELISA developed by Cheng et al using serum from 32 melioidosis patients and 20 healthy donors from Malaysia showed a sensitivity of 93.7% with a specificity of 100% [23].

Antibodies to OPS are highly elevated in melioidosis patients. Analysis of a follow-up set of 200 serum samples from diabetic or non-diabetic melioidosis patients in this study showed sensitivity to be 79%, which was consistent with the high sensitivity result of our previous study (72%) [21]. We observed higher seropositivity for both Hcp1 and OPS in diabetic melioidosis patients compared to those without diabetes. This has been previously seen for the IHA in Northern Australia [39] and for IHA in our laboratory in Thailand (manuscript in preparation). One possible explanation for the high seropositivity observed in diabetic patient is the alteration in the balance between cell-mediated immunity and humoral immunity in response to *B*. *pseudomallei* infection, for example due to enhanced polyclonal B-cell stimulation in Type 2 diabetes secondary to chronic hyperactivation of the innate immune response [40]. Ongoing studies are exploring the causality and exact mechanisms of higher antibody titers to *B*. *pseudomallei* in patients with diabetes. We reported no significant difference in diagnostic sensitivity between bacteremic versus non-bacteremic patients and survivors versus non-survivors. Thus, Hcp1 and OPS appear to be two promising antigens for further development of POC serological tests for all melioidosis patients including diabetics.

The induction of antibody responses to Hcp1 in melioidosis patients is relatively rapid with our results showing high median anti-Hcp1 titers at week 0. Interestingly, these titers were decreased at week 12 and week 52 for patients who recovered from the disease. Based on this, it appears that antibody titers to Hcp1 may be a useful serodiagnostic marker for acute infections as well as for monitoring the disease progression or recovery. The induction of antibody to OPS appeared to be slower with OPS-specific antibodies detectable at week 12 but declining by week 52. Further studies will be necessary to determine when OPS-specific antibody titers reach peak levels.

Our experiments focused on follow-up patients highlights the variation of individuals in the direction and rate of antibody titer changes over time. These results provide evidence of inter-individual variation in responses to the same *B*. *pseudomallei* antigens which may involve specific immune statuses, variable past exposures, infecting bacterial strains, and clinical disease factors. We recognize that our serological tests using a single serum dilution (1:2,000) may be of relatively limited value for following disease progression and that determining endpoint antibody titers will be more useful for assessing the variability of antibody levels in melioidosis patients.

In conclusion, this study establishes that Hcp1 and OPS are useful targets for serodiagnosis of melioidosis in various groups of patients. Overall, the Hcp1-ELISA provided better diagnostic assay values than the OPS-ELISA. When used in non-endemic areas, a combined Hcp1/OPS-ELISA showed increased sensitivity compared to the ELISAs using Hcp1 or OPS alone. Our results support accelerated development of Hcp1-based assays for a much needed POC test for the diagnosis of acute melioidosis.

## Supporting information

S1 Fig Individual antibody titers to Hcp1 in serum samples of 103 melioidosis patients who were survived within one year. The antibody titer was determined at week 0, week 12 and week 52 by ELISAs using cut-off titers at specificity 95%. A, serum no. 1203-M002 to 1203-M031; B, serum no. 1203-M034 to 1203-M075; C, serum no. 1203-M077 to 1203-M120; D, serum no. 1203-M121 to 1203-M162; E, serum no. 1203-M163 to 1203-M199.
(TIF)

S2 Fig Individual antibody titers to OPS in serum samples of 103 melioidosis patients who were survived within one year. The antibody titer was determined at week 0, week 12 and week 52 by ELISAs using cut-off titers at specificity 95%. A, serum no. 1203-M002 to 1203-M031; B, serum no. 1203-M034 to 1203-M075; C, serum no. 1203-M077 to 1203-M120; D, serum no. 1203-M121 to 1203-M162; E, serum no. 1203-M163 to 1203-M199.
(TIF)
