# Supplementary figures and images for: Comparison of O-polysaccharide and hemolysin co-regulated protein as target antigens for serodiagnosis of melioidosis
Source: PLoS Negl Trop Dis. 2017 Mar 30;11(3):e0005499. doi: 10.1371/journal.pntd.0005499 (PMC5395236; doi:10.1371/journal.pntd.0005499)

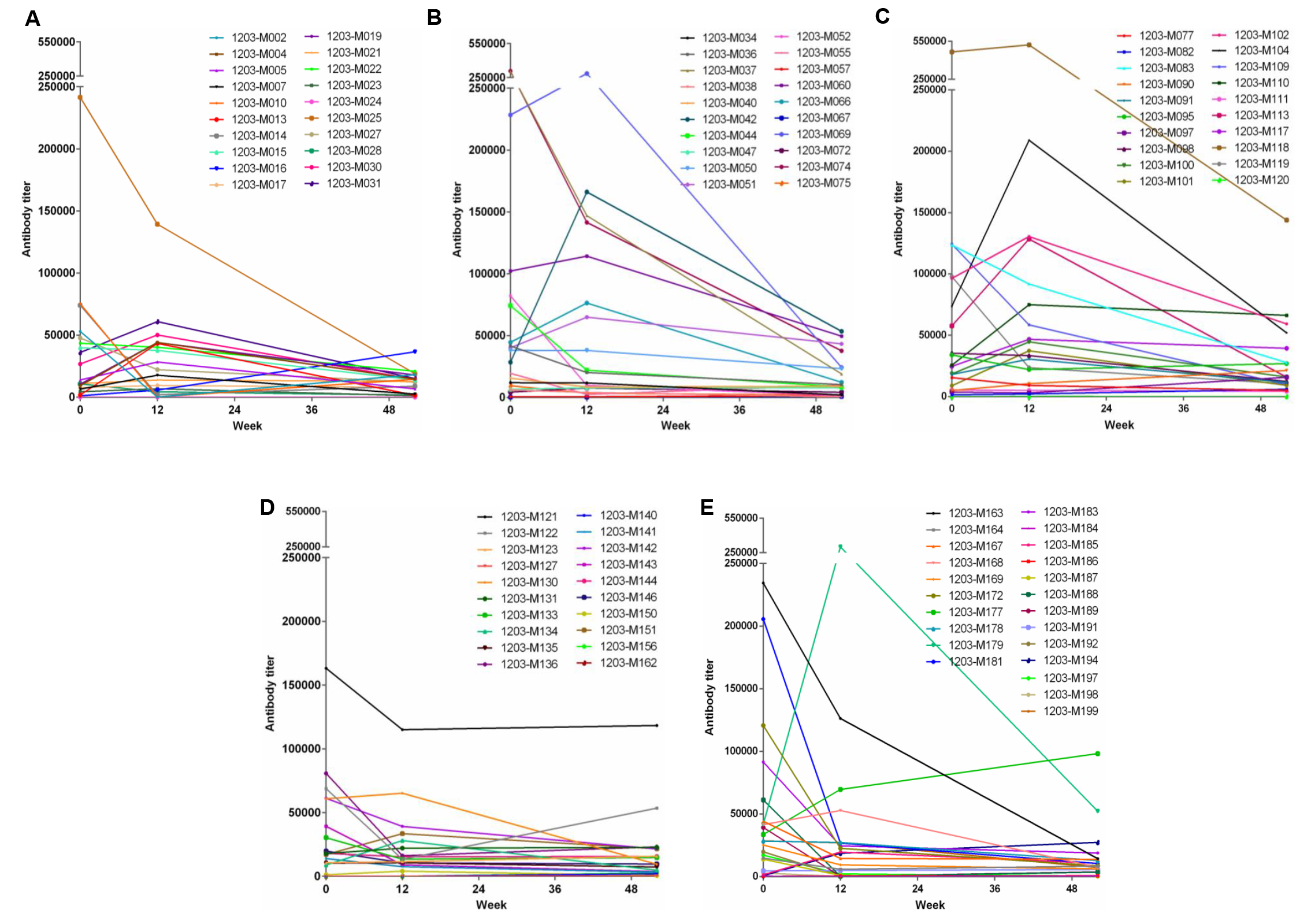

Supplement: S1 Fig — The antibody titer was determined at week 0, week 12 and week 52 by ELISAs using cut-off titers at specificity 95%. A, serum no. 1203-M002 to 1203-M031; B, serum no. 1203-M034 to 1203-M075; C, serum no. 1203-M077 to 1203-M120; D, serum no. 1203-M121 to 1203-M162; E, serum no. 1203-M163 to 1203-M199. (TIF) [file pntd.0005499.s001.tif]

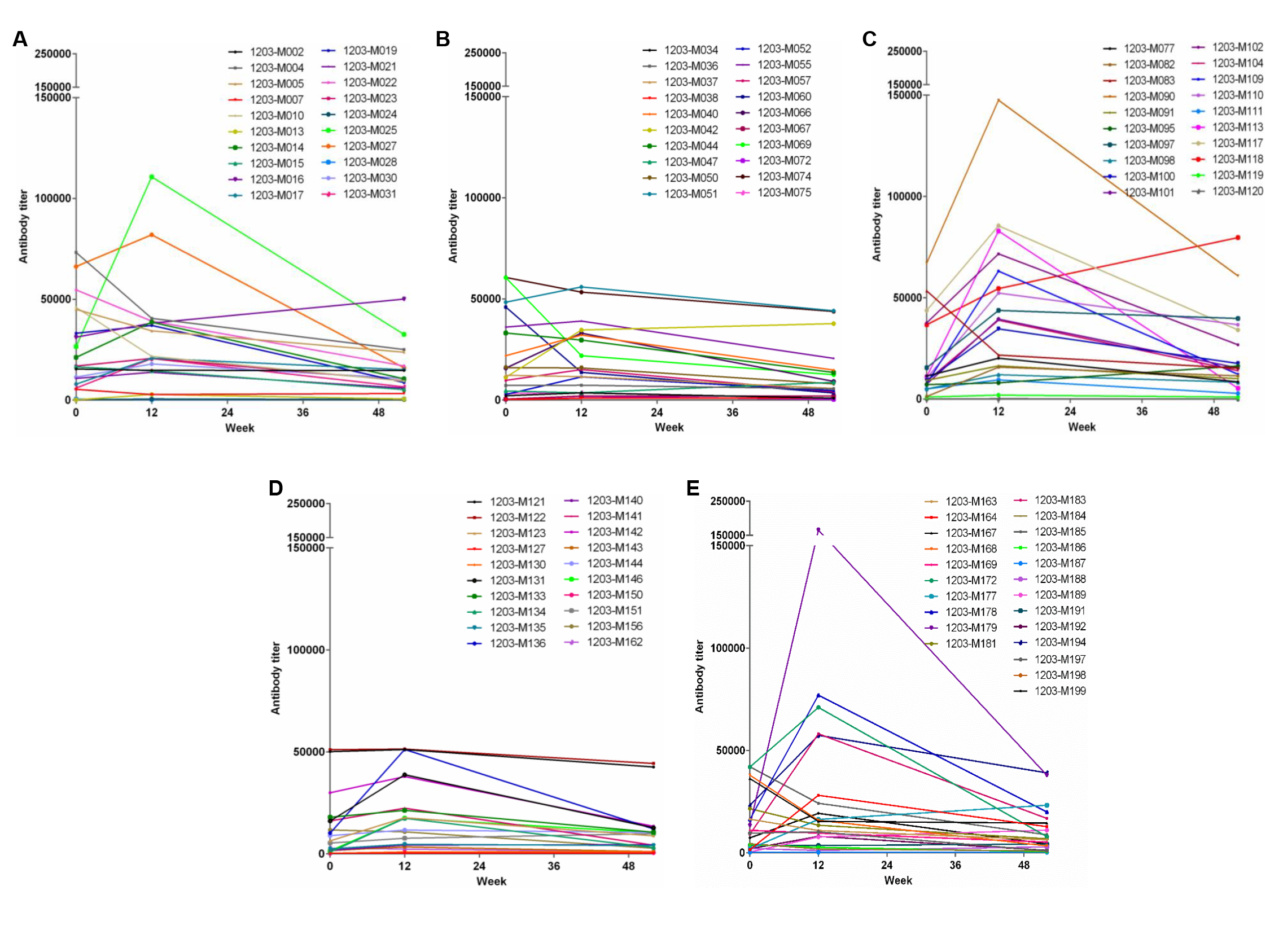

Supplement: S2 Fig — The antibody titer was determined at week 0, week 12 and week 52 by ELISAs using cut-off titers at specificity 95%. A, serum no. 1203-M002 to 1203-M031; B, serum no. 1203-M034 to 1203-M075; C, serum no. 1203-M077 to 1203-M120; D, serum no. 1203-M121 to 1203-M162; E, serum no. 1203-M163 to 1203-M199. (TIF) [file pntd.0005499.s002.tif]
